# Editorial: Women in cancer molecular targets and therapeutics, volume II: 2022

**DOI:** 10.3389/fonc.2023.1241793

**Published:** 2023-07-07

**Authors:** Ioana Berindan-Neagoe, Cinzia Lanzi

**Affiliations:** ^1^ Research Center for Functional Genomics, Biomedicine and Translational Medicine, University of Medicine and Pharmacy “Iuliu Hatieganu”, Cluj-Napoca, Romania; ^2^ Molecular Pharmacology Unit, Department of Experimental Oncology, Fondazione IRCCS Istituto Nazionale dei Tumori, Milan, Italy

**Keywords:** drug resistance, immunotherapy, metastasis, microbiome, tumor microenvironment

The second volume of *Women in Cancer Molecular Targets and Therapeutics* presents 22 articles authored by woman scientists, including original researches, reviews, hypothesis and theory, and a clinical trial across different fields of Oncology. Welcoming the success of this Edition, we would like to quote and share the words of the general director of UNESCO, Audrey Azoulay, “*By advancing the cause of women, we can drive progress in science*”.

Drug resistance has been the main theme addressed in the Research Topic. The development of resistance to therapy remains a major obstacle to the achievement of cures in patients with cancer. As new mechanisms are emerging, studies to identify markers predicting response to therapy and drivers of treatment resistance prompt novel approaches to overcome resistance (1). Pelullo et al. describe a new mechanism of intrinsic resistance to the standard adjuvant treatment, 5FU/OXP, in colorectal cancer (CRC) cells overexpressing Jagged1, the Notch-ligand involved in *KRAS*-mutated CRC progression. By inducing Jagged1 processing/activation through ERK1/2 activation, 5FU and OXP treatments promoted the accumulation of the Jag-ICD intracellular domain, a proteolytic oncogenic target of the KRAS signaling pathway, resulting in the selection of a drug-resistant subpopulation. Jagged1 overexpression is thus proposed as a potential predictive biomarker of chemotherapy outcome.

Acquired resistance is a major hurdle limiting the benefit also in patients treated with targeted therapies. With the goal to investigate MAP kinase pathway-independent mechanisms of resistance to BRAF/MEK inhibitors in *BRAF*-mutant melanoma cells, Reddi et al. applied a new approach to identify changes in the chromatin state affecting gene expression and signaling pathways. Through integrative analysis combining RNA-seq and assay for transposase-accessible chromatin with high-throughput sequencing (ATAC-seq), they discovered global changes in chromatin accessibility regions resulting in changes of mRNA expression of both known and new genes which might be further investigated as candidate resistance biomarkers.

The contribution of the tumor microenvironment (TME) to tumor growth, progression and resistance evolution against different therapeutic strategies, is increasingly recognized. In a review addressing cancer resistance to photodynamic therapy, Cerro et al. focus on the role of cancer associated fibroblasts and macrophages as well as of cytokines generated by these tumor stromal cells in modulating the outcome of photodynamic therapy in non-melanoma skin malignant lesions. In addition, they also summarize strategies investigated to prevent resistance to this therapeutic modality which has much grown in recent years. Zuo et al., in their analysis conducted in tumor samples of advanced epithelial ovarian cancer (EOC), confirmed the association of high expression of poly (ADP-ribose) polymerase 1 (*PARP1)* with platinum-resistance and poor prognosis. However, the study showed that PARP1 protein overexpression in the tumor surrounding stromal cells led to opposite effects. These findings suggest that the stromal PARP1 may modulate EOC progression and platinum sensitivity, eventually affecting even the response to PARP1 inhibitors (PARPi) currently used after the first-line platinum-based therapy. PARPi treatments have shown great promise in homologous recombination (HR)-deficient tumors. However, initial enthusiasm has been dampened by the high rate of drug resistance involving impairment of the DNA repair system and cell cycle progression. Jiang et al. show that PARPi resistance mediated by recovery of DNA repair *via* activation of the AKT/FOXO3a/GADD45a pathway could be blocked in different cancer cell lines by a combined targeting of PARP1 and the redox enzyme NAD(P)H:quinine oxidoreductase 1 (NQ01).

Intrinsic chemotherapy resistance, and a peculiar TME counteracting the effects of therapies, contribute to poor survival rate in pancreatic cancer patients. Immunotherapy using checkpoint blockade currently represents a treatment option for only a small subset of patients, while several immunotherapeutic approaches are pursued in clinical trials. Oncolytic virus therapy exploits the capability of viruses to infect cancer cells inducing the release of tumor antigens in the blood and activating anticancer immune response. Preclinical and clinical studies addressing this new type of immunotherapy for pancreatic cancer are reviewed by Nisar et al. They also describe new approaches to promote antitumor response and improve safety profile using oncolytic viruses and their combination with chemotherapeutics. In this regard, the same authors (Nisar et al.), in a study aimed at optimizing the virus tumor selectivity, analyzed the binding affinity and interaction profile of the L5 virus coat protein of three adenovirus serotypes with pancreatic cancer cell surface receptors. Scanlan et al. focus on the use of Herpes Simplex Virus 1 (HSV-1), discussing advantages and limitations of talimogene laherparevec (T-VEC), the only clinically approved oncolytic cancer viral therapy, as well as approaches currently being developed for improvement of safety and efficacy.

Cancer stem cells, unresponsive to conventional therapies are supposed to support tumor recurrence. Therefore, treatments able to affect these cells would provide a chance to circumvent tumor resistance. Pavlova et al. propose, as an alternative approach to cytotoxic treatment of glioblastoma, a differentiation therapy which, by stimulating the maturation of tumor cells would deprive them of their proliferative potential. Searching for effectors able to induce neural differentiation of human glioblastoma cells, they found that a stepwise treatment with a G-quadruplex pseudo-aptamer followed by neuro-inducer effectively inhibited glioblastoma cell proliferation also blocking division of CD133+ stem-like cells.

In research aimed at developing new efficient anticancer drugs and overcoming limitations in drug resistance and side effects, Molinaro et al. identified a copper (II) indenoisoquinoline complex, WN197, which induced cell cycle arrest and autophagic cell death in breast, cervix and colon cancer cell lines by a mechanism of action based on inhibition of topoisomerases.

Immunotherapy has shown great promise in certain cancer indications improving patient’s standard of care, quality of life and survival. Nonetheless, barriers such as an immunosuppressive TME, inefficient trafficking, and tumor antigen heterogeneity limit the success of immunotherapy in solid tumors (2). A better understanding of the interactions between tumor and immune response will be critical to extend the impact of immunotherapy to more solid tumor types. A few studies in this Research Topic investigate approaches to improve immunotherapy outcomes in patients with specific solid tumor types. Hintzen et al. review therapeutic strategies directed against epidermal growth factor receptor (EGFR)-positive malignancies describing alternative immunotherapeutic approaches potentially overcoming limitations of current therapies. Focusing on AFM24, an EGFR/CD16A bispecific innate cell engager that binds to natural killer (NK) cells and macrophages, they discuss potential advantages over T cell-based therapies, or those targeting EGFR activity, in terms of toxicity profile and possibility of combinations with other treatment modalities.

Li et al. investigated the relationship between serum levels of androgens and immune checkpoint receptor expression on T cells in patients with breast cancer. Androgens levels were found lower than those in healthy controls and positively correlated with PD-1 expression on Vδ1^+^ T cells in patients with the luminal B and HER2 tumor subtypes, suggesting a potential benefit of combining androgens with PD-1 inhibitor targeted therapy.

By analyzing the expression levels of the ADAM (a disintegrin and metalloprotease) family members in glioma tissues and cell lines, Qi et al. observed an upregulation of ADAMDEC1. High expression of this ADAM was associated with immune cell infiltration and single-cell sequencing analysis indicated co-expression with matrix metalloproteinases, suggesting a potential role as a clinical biomarker and immunotherapy target.

The metastatic process, which remains the primary cause of death in cancer patients (3), has been addressed in this Research Topic under various aspects in mechanistic and therapeutic studies. In a bioinformatic analysis of publicly available datasets, Wang et al. found that levels of X-linked inhibitor of apoptosis protein (XIAP) mRNA were significantly higher in lung adenocarcinoma with brain metastasis (LUAD-BM) compared to LUAD without metastasis. A regulatory network of competing endogenous RNAs is suggested to upregulate XIAP, which could have a role as therapeutic target in LUAD-BM.

MicroRNA-31 (miR-31) acts as a tumor suppressor and its loss has been associated with high risk of metastases in breast cancer. Tian et al. identified miR-31 as a main target of potassium piperonate (GBK), a traditional antihyperlipidemic medicine in China and preclinical anti-cancer agent. Investigating the anti-invasive mechanism of GBK, they observed that miR-31, and its host gene LOC554202, were upregulated following treatment of breast cancer cells. Meanwhile, the expression of miR-31 target genes associated with cell migration and metastasis was downregulated.

There is an increasing effort towards the generation of preclinical models that recapitulate the metastatic process allowing drug screening and efficacy prediction. The recognized role of an additional microRNA, miR-10b, as a metastasis driver prompted investigation of a strategy to treat metastatic breast cancer based on its inhibition. Savan et al. showed similar histological features and miR-10b expression in human and feline mammary carcinoma (FMC). In an investigation aimed at bridging previous studies performed in mice to humans, they propose the applicability of spontaneous metastatic FMC as a translational model for preclinical testing of MN-anti-miR10b, a miR-10b antagomiR conjugated to iron oxide nanoparticles. De Angelis et al. describe a new orthotopic metastatic patient-derived xenograft (ortho-PDX) model of CRC. Preliminary evidence indicated that a combination of stemness and epithelial-to-mesenchymal transition traits of primary tumors, indicative of increased tumor aggressiveness, might be crucial for ortho-PDX engraftment and metastatization in mice, while metastasis-derived organoids lost mesenchymal features and acquired increased chemoresistance. These observations supported ortho-PDX as a faithful model of human metastatic CRC.

Two studies in the Research Topic addressed the complex regulation of proteins displaying a double role as tumor suppressor or oncogene. Fontana et al. examined the literature describing the role in different tumor types of ARID1A, a component of the mammalian SWI/SNF chromatin remodeling complex mutated in about 10% of human cancers, and discuss current strategies exploiting synthetic lethality for treating ARID1A deficient tumors. The *TP63* gene encodes two protein variants, TAp63 which has tumor suppressor activities, and ΔNp63 which promotes tumorigenesis and drug resistance. Pokorna et al. investigated DNA methylation as a potential epigenetic regulation of *TP63* gene transcription explaining tissue-specific expression pattern of the two p63 isoforms and their dysregulation in squamous cell carcinomas (SSC). Available evidence indicated that the DNA demethylation agent decitabine induced an increase in TAp63 and a concomitant reduction of ΔNp63 potentially switching the protein function from oncogenic to tumor suppressive in SSC cells. The authors propose two alternative hypotheses for the reciprocal isoform regulation.

Emerging evidence implicates host-intrinsic microorganisms and their genes (the microbiome) in the regulation of the tumor microenvironment affecting tumor development and progression as well as response to treatment (4). Richardson et al. review the current literature on the skin microbiome in cancer with a focus on the potential role in treatment-related skin toxicities. They comment on approaches to prevent and treat such toxicities by modulating the skin microbiome eventually improving patient’s quality of life.

Bone marrow transplant (BMT) is a treatment option in oncologic patients who need high-dose chemotherapy and/or radiation therapy. Examining the literature data about the role of the β3-adrenergic receptor (β3-AR) in the bone marrow, Nastasi et al. speculate that targeting β3-AR may support the homing and differentiation of hematopoietic stem cells in the bone marrow providing a useful support to overcome complications related to BMT.

Tang et al. report the results of a single-arm trial of the antiangiogenic multikinase inhibitor anlotinib in 42 patients with recurrent or metastatic malignant bone tumor. The drug demonstrated promising antitumor activities as second or later line of therapy and a manageable safety profile which warrant further investigation in randomized studies.

The two Editions of *Women in Cancer Molecular Targets and Therapeutics* reflect the variety of research performed by female scientists and highlight the huge contribution they are making in advancing the fight against cancer. These Research Topics renew the hope that gender equality will be even more pursued in Science.

We wish to thank all the authors, women and co-authors, for the contributions here collected and for sharing their findings and views on various aspects of cancer research.

## Author contributions

All authors listed have made a substantial, direct, and intellectual contribution to the work and approved it for publication.
